# Factors associated with bone health status of Malaysian pre-adolescent children in the PREBONE-Kids Study

**DOI:** 10.1186/s12887-021-02842-6

**Published:** 2021-09-03

**Authors:** Chung Yuan Chang, Kanimolli Arasu, Soon Yee Wong, Shu Hwa Ong, Wai Yew Yang, Megan Hueh Zan Chong, Meenal Mavinkurve, Erwin Jiayuan Khoo, Karuthan Chinna, Connie Marie Weaver, Winnie Siew Swee Chee

**Affiliations:** 1 Department of Nutrition & Dietetics, School of Health Sciences, International Medical University, No. 126, Jalan Jalil Perkasa 19, Bukit Jalil, 57000 Kuala Lumpur, Malaysia; 2 Department of Paediatrics, School of Medicine, International Medical University, Jalan Rasah, Negeri Sembilan, 70300 Seremban, Malaysia; 3 Faculty of Health & Medical Sciences, School of Medicine, Taylor’s University, No 1, Jalan Taylor’s, 47500 Subang Jaya, Selangor Malaysia; 4 Purdue University, West Lafayette, Indiana, USA

**Keywords:** Bone mineral density, Body composition, Vitamin D, Calcium, prepubertal, Malaysia

## Abstract

**Background:**

Modifiable lifestyle factors and body composition can affect the attainment of peak bone mass during childhood. This study performed a cross-sectional analysis of the determinants of bone health among pre-adolescent (*N* = 243) Malaysian children with habitually low calcium intakes and vitamin D status in Kuala Lumpur (PREBONE-Kids Study).

**Methods:**

Body composition, bone mineral density (BMD), and bone mineral content (BMC) at the lumbar spine (LS) and total body (TB) were assessed using dual-energy X-ray absorptiometry (DXA). Calcium intake was assessed using 1-week diet history, MET (metabolic equivalent of task) score using cPAQ physical activity questionnaire, and serum 25(OH) vitamin D using LC-MS/MS.

**Results:**

The mean calcium intake was 349 ± 180 mg/day and mean serum 25(OH)D level was 43.9 ± 14.5 nmol/L. In boys, lean mass (LM) was a significant predictor of LSBMC (β = 0.539, *p* < 0.001), LSBMD (β = 0.607, *p* < 0.001), TBBMC (β = 0.675, *p* < 0.001) and TBBMD (β = 0.481, *p* < 0.01). Height was a significant predictor of LSBMC (β = 0.346, *p* < 0.001) and TBBMC (β = 0.282, *p* < 0.001) while fat mass (FM) (β = 0.261, *p* = 0.034) and physical activity measured as MET scores (β = 0.163, *p* = 0.026) were significant predictors of TBBMD in boys. Among girls, LM was also a significant predictor of LSBMC (β = 0.620, *p* < 0.001), LSBMD (β = 0.700, *p* < 0.001), TBBMC (β = 0.542, *p* < 0.001) and TBBMD (β = 0.747, *p* < 0.001). Calcium intake was a significant predictor of LSBMC (β = 0.102, *p* = 0.034), TBBMC (β = 0.122, *p* < 0.001) and TBBMD (β = 0.196, *p* = 0.002) in girls.

**Conclusions:**

LM was the major determinant of BMC and BMD among pre-adolescent Malaysian children alongside other modifiable lifestyle factors such as physical activity and calcium intake.

## Background

Prepubertal age is an important period in life for rapid growth and bone accretion leading to peak bone mass attainment during adolescence and early adulthood. Accumulation of bone mass during this rapid growth phase is important for the prevention of osteoporosis at adulthood [1]. A 10 % increase in peak bone mass is estimated to halve the risk of an osteoporotic fracture in adult life [2].

Genetics may determine approximately 80 % of bone mineral density (BMD) acquisition during childhood [3], however, modifiable factors including nutrition, physical activity and body composition are estimated to affect up to 20 % of BMD [4, 5]. A National Osteoporosis position paper, which included a systematic review of all lifestyle factors influencing development of peak bone mass, concluded that evidence was sufficient to achieve an A grade (strongest evidence with consistent findings from multiple representative studies) for only calcium intake and physical activity [6]. Calcium is the major constituent of bone mineral and increasing dietary calcium towards recommended intakes suppresses bone resorption. Physical activity and exercise exert a continuous stimulus on bone as a living tissue that responds to mechanical load, and therefore, is essential to maintain a normal bone mass [7]. An adequate level of Vitamin D status received a B grade (moderate) level of evidence [6]. Adequate vitamin D status facilitates calcium absorption by the vitamin D-dependant pathway, more dominant when calcium intake is low, necessary for normal calcification of the growth plate and the mineralisation of bones.

Body weight, a genetically determined factor that is also modifiable, is one of the strongest predictors of bone mass [6]. Between the two main components of body weight, lean mass (LM) and fat mass (FM), it remains uncertain which one exerts a greater effect on bone mass accretion during puberty. In a systematic review by Sioen and colleagues [8], LM consistently showed a significant positive association with BMD and bone mineral content (BMC). The role of body fat on bone acquisition is contradictory and may depend on the nature of the fat (amount and distribution) as well as sex and pubertal status.

While the burden of osteoporotic fractures is markedly increasing around the world [9], the greatest impact is expected to occur in Asia with Malaysia being projected to have the highest increase of up to 3.55-fold in hip fractures by the year 2050 due to a rapidly ageing population [10]. Osteoporosis is often called a childhood disease because building peak bone mass occurs in childhood [3]. Although it is widely reported that Asian children have habitually low calcium intakes and a high prevalence of vitamin D deficiency [11, 12], a systematic review revealed that there are limited Asian studies examining the association of these conditions with BMD attainment [6]. Malaysian pre-adolescent children have been reported to have calcium intakes below 500 mg/day, low physical activity levels and low serum vitamin D status [12–15]. In addition, Malaysia has the second-highest rate of childhood obesity in South East Asia with a prevalence of 16.5 % in children aged 8–12 years old [16]. Therefore, a better understanding of the role of body composition on skeletal health and factors associated with low LM and high FM become important. This study performed a cross-sectional analysis on the determinants of bone health among pre-adolescent Malaysian children with habitual low calcium intakes and low vitamin D status from the baseline data of the PREBONE-Kids Study. We hypothesised that modifiable lifestyle factors such as calcium and vitamin D intakes, physical activity and body composition would predict the bone health status of these children.

## Methods

### Study design and participants

The PREBONE-Kids Study is a 1-year randomised, double-blind, placebo-controlled trial of soluble corn fiber (SCF) on bone indices in pre-adolescent primary school children residing in Kuala Lumpur (ClinicalTrials.gov identifier: NCT03864172). We recruited 243 school children aged 9 to 11 years (127 boys and 116 girls) the 1-year study during the period of March 2017 through March 2018. The study included participants who were healthy as determined by a standard medical assessment, Tanner Stage 1 or 2 based on breast development for girls and pubic hair in boys, premenarcheal for girls and able to provide assent. Participants were excluded if they had a history of serious medical conditions and received therapy with medications known to interfere with bone metabolism (e.g. steroids, hormones, diuretics, cortisone or anti-seizure medication). Ethical approval for the study was obtained from the Research and Ethics Committee of the International Medical University (IMU) (Trial no: R182/2016). Informed consent was obtained from parents or legal guardians and assents were obtained from the participants. Details of the PREBONE-Kids study protocol was published previously [17].

### Baseline examinations

Anthropometry measurements were taken by trained research assistants following the International Society for the Advancement of Kinanthropometry (ISAK) standard procedures [18]. The height was measured using vertical stadiometer (SECA 206, Hamburg, Germany) to the nearest 0.1 centimetres (cm) and weight was measured using a portable digital scale (Tanita HD-301, Tanita Corporation, Japan) to the nearest 0.1 kg (kg). Body mass index (BMI) values were computed as the ratio between weight (kg) and the square of height (meter). In this study, standardized BMI values based on World Health Organization were used to classify the participants into four BMI categories; thin (BMI Z-score < -2.0), normal (≥ -2.0, ≤ 1.0), overweight (BMI Z-score > 1.0, ≤ 2.0) and obese (BMI Z-score > 2.0) [19].

Total body and lumbar spine bone density and total body composition were measured using GE Lunar iDXA (GE Healthcare, USA) with paediatric software (Lunar enCORE version 13.60.033) using population references for Asian children [20]. The dual-energy X-ray absorptiometry (DXA) scans provided measurements of BMC and BMD for the total body (TBBMC and TBBMD) and lumbar spine L1-L4 (LSBMC and LSBMD) as well as LM, FM and percent body fat (BF%). The coefficient of variation (CV%) of the phantom was 0.35 %. Imaging technician’s CV% for TBBMD, LSBMD, LM and FM were 0.42 %, 0.83 %, 1.37 and 0.86 % respectively. These measurements were performed within one week of data collection of questionnaires and anthropometry at the schools.

Participants were asked using a structured interview method about their habitual food intake in terms of meal patterns, types of foods consumed and frequency using a 7-days diet history form. Portion sizes were estimated using household measurements with the assistance of a food portion album and these were verified with their parents or caretakers. The portions consumed were then converted to grams and analysed for calcium and vitamin D content using Nutritionist Pro Diet Analysis Software (version 7.4.0, 2019, Axxya Systems, LLC, USA) in which Nutrient Composition of Malaysian Foods (Tee et al., 1997) was the primary data source. Alternatively, for foods that were not available in the Malaysian food database, the Singapore Energy and Nutrient Composition of Food [21] was used. In addition, nutrient labels were used for manufactured food products and beverages.

Physical activity level (PA) was measured using a physical activity questionnaire (cPAQ Malay version) which has been validated among Malaysian children [22]. The questionnaire consisted of 3 sections: habitual activities (transportation, school activities, extra-curriculum, sport and club activities), leisure activities and housework. Metabolic equivalent task (MET) score was calculated based on Ainsworth et al. [22, 23]. and Kemper et al. [24].

Non-fasting blood samples were collected for serum 25(OH)D analysis on the same day as the questionnaires and anthropometry data collection. Serum samples were extracted through centrifugation at 1500–2000 g for 10 min at 4 °C. The serum samples were then stored at -80 °C in an upright position until analysis for 25(OH)D. All vitamin D metabolites were analysed by liquid chromatography-tandem mass spectrometry (LC-MS/MS) with an Agilent 1260 Infinity liquid chromatograph (Agilent Technologies, Waldbronn, Germany) coupled to a QTRAP® 5500 tandem mass spectrometer (AB SCIEX, Foster City, CA, USA) using a MassChrom® 25(OH)Vitamin D3/D2 in serum/plasma reagent kit including a 3-epi-25(OH)Vitamin D3/D2 upgrade diagnostics kit (Chromsystems, Munich, Germany). All analyte values of the calibrator and control were traceable to certified substances and standard reference materials of the National Institute of Standards and Technology. The coefficients of variation of serum 25(OH)D3, 25(OH)D2, and 3-epi-25(OH)D3 were 5.9 %, 3.3 and 4.6 % respectively.

### Statistical analysis

The distribution of variables were assessed based on skewness and kurtosis values [25]. Quantitative variables were described as either medians and ranges or means ± standard deviation (SD). Independent sample t-test was used to examine the mean differences in the quantitative variables between boys and girls. Qualitative variables were reported as frequencies and percentage. Multiple linear regression analysis was used to determine significant predictors of BMD and BMC at the lumbar spine and total body. Multicollinearity was tested and in the final model, only variables that were significant in the stepwise analysis were considered. All calculations were performed using Statistical Package for the Social Sciences (SPSS) version 21.0 for Windows. In all tests, a *p*-value of less than 0.05 was statistically significant.

## Results

The descriptive characteristics of the participants are shown in Table 1. The participants were predominantly Malays (90.5 %) followed by Indians (9.5 %). The mean age was 10.1 ± 1.0 years. Most of the participants were in Tanner Stage 1 (95 %) while a small percentage were in Tanner Stage 2 (5 %).

**Table 1:** Baseline characteristics of participants from PREBONE-KIDS study (*N*=243**)**

	Total (***n***=243)	Boys (***n***= 127)	Girls (***n***=116)	***p-***value*
Age	10.1 ± 1.0	10.2 ± 0.9	10.0 ± 1.0	0.122
Ethnicity				
Malay	220 (90.5)	110(86.6)	110 (94.8)	
Indian	23 (9.5)	17 (13.4)	6 (5.2)	
Tanner stage				
Stage 1	230 (94.7)	125 (98.4)	105 (90.5)	
Stage 2	13 (5.3)	2 (1.6)	11 (9.5)	
Weight (kg)	34.0 ± 12.1	34.7 ± 13.3	33.1 ± 10.8	0.292
Height (cm)	135.8 ± 9.1	136.1 ± 8.9	135.4 ± 9.5	0.580
BMI (kg/m^2^)	18.0 ± 4.5	18.3 ± 4.8	17.7 ± 4.2	0.332
BMI-for-age Z-score	0.187 ± 1.693	0.321 ± 1.742	0.040 ± 1.632	0.198
BMI Z-score classification, n (%)				
Thinness	21 (8.7)	9 (7.1)	12 (10.3)	
Normal	142 (58.4)	74 (58.3)	67 (57.8)	
Overweight	37 (15.2)	18 (14.2)	21 (18.1)	
Obese	43 (17.7)	26 (20.5)	16 (13.8)	
Bone parameters				
Lumbar Spine (LS)				
Area (cm^2^)	29.7 ± 4.2	30.0 ± 4.1	29.3 ± 4.3	0.182
BMC (g)	21.7 ± 5.2	21.6 ± 4.7	21.9 ± 5.7	0.725
BMD (g/cm^2^)	0.725 ± 0.091	0.715 ± 0.081	0.736 ± 0.100	0.064
Total Body (TB)				
Area (cm^2^)	1462.0 ± 184.6	1480.5 ± 188.3	1441.8 ± 179.0	0.102
BMC (g)	1129.5 ± 231.6	1160.4 ± 237.9	1095.6 ± 220.6	0.029
BMD (g/cm^2^)	0.768 ± 0.075	0.780 ± 0.075	0.754 ± 0.072	0.006
BMD z-score	0.789 ± 0.960	0.890 ± 0.921	0.678 ± 0.994	0.087
Total body less head (TBLH)				
Area(cm^2^)	1234.5 ± 183.7	1251.0 ± 187.8	1216.5 ± 178.2	0.143
BMC(g)	815.5 ± 220.3	836.6 ± 231.3	792.4 ± 206.2	0.118
BMD (g/cm^2^)	0.650 ± 0.086	0.658 ± 0.088	0.642 ± 0.082	0.140
Body composition				
LM (kg)	21.79 ± 5.29	22.50 ± 5.40	21.00 ± 5.07	0.026
FM (kg)	10.93 ± 7.08	11.00 ± 7.98	10.85 ± 5.98	0.876
BF (%)	29.87 ± 8.44	28.82 ± 9.17	31.06 ± 7.39	0.035
Serum 25(OH)D (nmol/L)	43.9 ± 14.5	50.3 ± 13.7	36.8 ± 11.9	<0.001
Energy (Kcal)	1457 ± 450 (IQR: 1129 - 1711)	1543 ± 463 (IQR: 1213 - 1778)	1363 ± 417 (IQR: 1071 - 1574)	0.002
Protein intake	61.4 ± 21.6 (IQR: 48.2 – 69.7)	65.7 ± 23.3 (IQR: 50.8 – 72.8)	56.6 ± 18.7 (IQR: 44.0 – 66.6)	0.001
Calcium intake (mg)	349 ± 180 (IQR: 218 – 459)	356 ± 166 (IQR: 217 – 468)	341 ±194 (IQR: 218 – 452)	0.510
Vitamin D (μg)	1.5 ± 1.6 (IQR: 0.3 – 2.2)	1.5 ± 1.6 (IQR: 0.4 – 2.3)	1.4 ± 1.6 (IQR: 0.2 – 2.1)	0.638
PA level (MET scores)	822 ± 447	961 ± 502	670 ± 317	<0.001

*Abbreviation*: *BMI* body mass index, *LM* lean mass, *FM* Fat mass, *BF* body fat, *25(OH)D* 25 hydroxyvitamin D, *PA* physical activity, *IQR* interquartile range, *MET* metabolic equivalent task

* Student’s t-test and *p*<0.05 considered significant

There were no significant differences between males and females in the mean weight, height and BMI. Among the participants, 15.2 % were overweight and 17.7 % were obese. Among the boys, 9 (7.0 %) were thin, 76 (59.4 %) were normal weight, 16 (12.5 %) overweight and 27 (21.1 %) obese. Among the girls, 12 (10.4 %) were thin, 66 (57.4 %) were normal weight, 21 (18.3 %) overweight and 16 (13.9 %) obese. Although boys and girls had similar fat mass, the proportion of fat to body weight as measured by BF% was higher among the girls (31.06 ± 7.39 %) compared to the boys (28.82 ± 9.17 %, *p* = 0.035). The LM was higher among the boys (22.50 ± 5.40 kg) compared to the girls (21.00 ± 5.07 kg, *p* = 0.026). The TBBMC (boys: 1160.4 ± 237.9 g vs. girls: 1095.6 ± 220.6 g, *p* = 0.029) and TBBMD (boys: 0.780 ± 0.075 g/cm^2^ vs. girls: 0.754 ± 0.072 g/cm^2^, *p* = 0.006) were significantly higher in boys compared to girls. There were no significant sex differences in LSBMC, LSBMD, bone area or BMD Z-scores.

The mean calcium intake for all participants was349 ± 180 mg/day (range: 218 – 459 mg/day) which met only about 25% of the recommended calcium intake for Malaysian children in this age-group. The calcium intakes were equally low in both boys and girls. Protein intake was significantly higher among boys than girls (65.7± 23.3 g vs 56.6 ± 18.7 g; *p* =0.001). In terms of physical activity MET scores, the boys were significantly more active than girls (boys: 961 ± 502 vs girls: 670 ± 317, *p* < 0.001). The overall mean serum 25(OH)D level was 43.9 ± 14.5 nmol/L. The level was significantly higher among the boys (boys: 50.3 ± 13.7 nmol/l vs girls: 36.8 ± 11.9 nmol/l, *p* < 0.001) compared to the girls.

The variables for the stepwise regression analyses included age, height, LM, FM, MET scores, 25(OH)D, protein and calcium intake. Results from stepwise regression analyses for boys and girls are shownin Table 2 and Table 3 respectively.

**Table 2:** Stepwise regression analysis for predictors of lumbar spine and total body BMD and BMC for boys (*N*=127)

Dependent variables	Significant predictors	Regression coefficients		*p-*value	R^2^
		Unstandardized (B)	Standardized (β)		
LSBMC	LM	0.0005	0.539	<0.001	0.732
	Height	0.184	0.346	<0.001	
	Constant	-13.973		<0.001	
LSBMD	LM	0.00001	0.607	<0.001	0.364
	Constant	0.510			
TBBMC	LM	0.030	0.675	<0.001	0.866
	Height	7.564	0.282	<0.001	
	Constant	-537.780			
TBBMD	LM	0.00001	0.481	<0.001	0.583
	FM	0.000002	0.261	0.034	
	MET score	0.00002	0.163	0.026	
	Constant	0.579			

The tested variables were age, height, lean mass, fat mass, MET scores, 25(OH)D, calcium and protein intake

*Abbreviation*s: *LS* lumbar spine, *TB* total body, *BMC* bone mineral content, *BMD* bone mineral density, *LM* lean mass, *FM* Fat mass, *MET* metabolic equivalent task

**Table 3:** Stepwise regression analysis for predictors of lumbar spine and total body BMD and BMC for girls (*N*=116)

Dependent variables	Significant predictors	Regression coefficient		*p-*value	R^2^
		Unstandardized (B)	Standardized (β)		
LSBMC	LM	0.001	0.620	<0.001	0.743
	Height	0.163	0.274	0.004	
	Calcium	0.003	0.102	0.034	
	Constant	-15.798		0.008	
LSBMD	LM	0.00001	0.700	<0.001	0.490
	Constant	0.446			
TBBMC	LM	0.024	0.543	<0.001	0.895
	Height	7.897	0.340	<0.001	
	FM	0.005	0.123	0.036	
	Calcium	0.139	0.122	<0.001	
	Constant	-566.616			
TBBMD	LM	0.00001	0.747	<0.001	0.573
	Calcium	0.00001	0.197	0.002	
	Constant	0.506			

The tested variables were age, height, lean mass, fat mass, MET scores, 25(OH)D, calcium and protein intake

*Abbreviations*: *LS* lumbar spine, *TB* total body, *BMC* bone mineral content, *BMD* bone mineral density, *LM* lean mass, *FM* Fat mass

Among the boys, the predictors for LSBMC were LM(β = 0.539, *p* < 0.001) and height (β = 0.346, *p* < 0.001) with an R-square value of 0.732. LSBMD was significantly predicted by only LM (β = 0.607, *p* < 0.001) and the R-square value was 0.364. The predictors for TBBMC were also LM (β = 0.675, *p* < 0.001) and height (β = 0.282, *p* < 0.001). The R-square value was 0.866. TBBMD was significantly predicted by LM (β=0.481, *p* < 0.001),FM (β = 0.261, *p* = 0.034) and MET Score (β = 0.163, *p* = 0.026) and the R-square value was 0.583.

Among the girls, LSBMC was significantly predicted by LM (β = 0.620, *p* = <0.001) and height (β = 0.274, *p* = 0.004) and calcium intake (β = 0.102, *p* = 0.034). The R-square value was 0.743. The predictor for LSBMD was only LM (β = 0.700, *p* < 0.001) with an R-square value of 0.490. TBBMC was significantly predicted by LM (β = 0.543, *p* < 0.001), height (b = 0.340, *p* < 0.001), calcium intake (b = 0.123, *p* < 0.001) and FM(β = 0.122, *p* = 0.036). The R-square value was 0.895. TBBMD was significantly predicted by LM (β = 0.747, *p* < 0.001) and calcium intake (β = 0.197, *p* = 0.002) with the R-square value of 0.573.

## Discussion

This is the first study in Malaysia and one thefew studies in Asian countries reporting the association between modifiable lifestyle factors and body composition on BMD and BMC in pre-adolescent children measured by DXA. Other studies have reported on determinants of Asian adolescent bone health status using bone ultrasound [26–28].

In this study, 15.2% of the participants wereoverweight and another 17.7% were obese.

These figures are reflective of the nationally reported prevalence of overweight and obesity, 14.4% and 20.1% respectively, for children aged 7-12 years old [16]. The prevalence of childhood obesity is alarmingly high in Malaysia as compared to other Asian countries as determined by a meta-analysis which reported that the pooled prevalence, overall for boys and girls aged 5-11 years, was only 5.8% [29].The sex differences observed in our study are also reflective of worldwide trends whereby boys are often reported to have a higher prevalence of obesity than girls [29].Our study reported a higher BF% in girls as opposed to boys that had a higher LM and TBBMD/BMC. These findings are typically observed in pre-adolescent children and influenced by hormonal effects and other phenomena [30].

This study also determined that LM was the strongest predictor of BMC and BMD of total body and lumbar spine in boys and girls, among other variables including FM, calcium intake, energy intake, protein intake, serum 25(OH)D and physical activity. Two published studies from India and Iran amongst adolescents (mean age ranged from 13.2-15.4 years old) reported a positive association between lean mass and bone parameters as measured by DXA [31, 32]. The effect of the association between LM with BMC and BMD (standardised β = 0.5to 0.8) were similar to the studies reported in Caucasian children of a similar age group [33–35]. Furthermore, our finding is aligned with the systematic review by Sioen et al. [8]which reported that LM was a stronger determinant of bone parameters as measured by DXA than FM and BF %. The effect of LM on bone mass has been attributed to the higher tensile force LM exerts on bone as explained by the mechanostat theory [36–39]. It has been reported that the production of insulin-like growth hormone factor-1 (IGF-1) exerts a positive effect on osteogenesis before menarche. Moreover, Interleukin 6 (IL-6) had shown an impact on bone metabolism, however, whether the impact is favourable to bone formation remains unclear [40–42].

Based on the regression analyses, the effect of FM on TBBMD and TBBMC is much less than for LM. Fat mass may exert an equivalent mechanostat function as LM [43, 44]but the stimulation of bone cells by FM is not as effective as LM. Given that almost 33% of the participants in our study were overweight and obese, it is interesting to observe that LM instead of FM was more beneficial for bone accrual in this population. Farr et al. reported that in young girls aged 8-13years old, FM is correlated with volumetric BMD, periosteal circumference and strength; however, this FM effect was significantly attenuated after adjustment for muscle/lean mass [45]. Muscle adiposity was reported to have anegative impact on metabolic function such as insulin resistance, and thus, maynegatively influence cortical bone geometry [46].Controlling muscle adiposity (e.g. fat-muscle ratio) in obesity may provide better understanding on the relationship between body adiposity and bone acquisition in growing children.

The present study confirmed that height is also a significant determinant of BMC in boys and girls at both the total body and lumbar spine. It is well documented that BMC and BMD measurements by DXA are affected by height [47].The participants in our study were experiencing rapid growth and as McCormacket al. reported, as the skeleton grows and expands, BMC also increases exponentially [48].

Physical activity of the male participants in our study as measured by MET scores was a significant predictor of TBBMD. Boys were found to be more physically active than girls and their MET score wasfound to be a significant contributor to TBBMD, but not in girls. Physical activity is known to influence bone health through a similar mechanism as LM whereby activation of the mechanosensitive cells, osteocytes, embedded within the bone, signal molecules to stimulate osteogenesis [6].

We demonstrated that physical activity was the main predictor for LM in boys but not girls. The literature supports that weight-bearing and ground reaction force (GRF) are important for bone growth [24, 49]. A systematic review highlighted the significant changes in bone structure (cortical thickness and bone area) in response to mechanical loading and muscle function [49]. Majority of the boys in our study were involved in moderate to vigorous sports activities with high GRF such asrunning (MET intensity = 7.7), hockey and handball (MET intensity = 6.0). In contrast, the girls were generally involved in sports activities with shorter duration and lower intensity such as aerobics exercise (MET intensity = 5.0) and dancing (MET intensity = 4.0)during physical education classes, although some activities were as great as that for boys, i.e. hockey.

Calcium intake was a significant predictor of TBBMD,TBBMC and LSBMC amongst girls in this study. The participants had an average calcium intake that only met one-quarter of the national recommended calcium intake of 1300 mg [50]for adequate growth and bone health. Low calcium intakes are correlated with low BMD in Asian children and exert a negative impact on growth and adult height [51, 52]. More studies are needed to verify this relationship in other Asian populations.

About 40% of the children in this cohort had inadequate serum 25(OH)D level (<40nmol/L) [53]. The girls had a significantly lower serum 25(OH)D status as compared to boys. This finding can be explained by the fact that majority of the girls in our study have lower exposure to sunlight due to their religious attire, which only leaves their face and hands exposed. Furthermore, the girls also had lower levels of physical activity as compared to boys, reflecting less outdoor activities and exposure to the sun. However, neither our study nor a similar study among preadolescent children in South Africa with low levels of serum vitamin D showed any association between bone parameters and levels of 25(OH)D [35].

The study had predominantly Malay ethnicity among the study participants, a previously understudied group. Future studies should include other ethnic groups to elucidate whether the findings are similar in Chinese and Indian children in Malaysia. A strength of the study was the analysis of serum vitamin D using LC-MS/MS which is considered the reference method and the use of DXA to determine bone parameters, but a limitation was that we were not able to determine volumetric bone mass, microarchitecture of bone, or the fat–bone relationship with respect to visceral, bone marrow, and muscular adiposity. The range of some parameters in this cohort including calcium intake,vitamin D status and bone indices may have been too narrow to determine their full effects on bone, though these parameters were representative of growing pre-adolescent Asian children.

## Conclusion

Our study is the first to report the associationof modifiable lifestyle factors and body composition on bone parameters measured by DXA among pre-adolescent children in Malaysia. We found that LM is the major determinant of BMC and BMD alongside other modifiable lifestyle factors such as physical activity and calcium intake. Encouraging physical activity, calcium intake and optimum diets that build lean body mass should be the focus for developing public health guidance to ensure optimal bone health status during rapid growth.

## Data Availability

The datasets used during the current study are available from the corresponding author on reasonable request.

## Abbreviations

BMD: Bone mineral density
BMC: Bone mineral content
LS: Lumbar spine
TB: Total body
DXA: Dual-energy x-ray absorptiometry
MET: Metabolic equivalent task
LC-MS/MS: Liquid Chromatography with tandem mass spectrometry
cPAQ: Children physical activity questionnaire
25(OH)D: 25 hydroxyvitamin D
LM: Lean mass
FM: Fat mass
BF%: Body fat percentage
ISAK: International Society for the Advancement of Kinanthropometry
BMI: Body mass index
CV%: Coefficient of variation
SD: Standard deviation
SPSS: Statistical package of social sciences
TBLH: Total body less head
IGF-1: Insulin growth factor 1
IL-6: Interleukin-6
GRF: Ground reaction force
